# Dietary inflammatory index and Mediterranean Diet in female reproductive health: a systematic review and meta-analysis of dietary inflammatory potential

**DOI:** 10.3389/fnut.2026.1904722

**Published:** 2026-08-11

**Authors:** Beatriz Mérida-Yáñez, Sofía García-Oliva, Irene Antúnez-Calvente, Nerea de la Torre-Pérez, Francisco Javier Muñoz-Vela, Juan Gómez-Salgado, Antonio Márquez-Díaz, Miguel Ángel Avellaneda-Roldán, Naore López-Gómez, Azahara Rúger-Navarrete

**Affiliations:** 1 Department of Nursing, Faculty of Health Sciences of Ceuta, Universidad de Granada, Granada, Spain; 2 Department of Obstetrics, Hospital Universitari Germans Trias i Pujol, Badalona, Spain; 3 Hospital Insular Nuestra Senora de los Reyes, Villa de Valverde, Spain; 4 Department of Medicine and Public Health, Faculty of Labour Sciences, University of Huelva, Huelva, Spain; 5 Safety and Health Postgraduate Programme, Universidad de Especialidades Espiritu Santo, Samborondón, Ecuador; 6 Department of Obstetrics, Hospital Regional Universitario de Malaga, Málaga, Spain

**Keywords:** endometriosis, fertility, Mediterranean Diet, ovarian function, polycystic ovary syndrome

## Abstract

**Introduction:**

Female reproductive health is increasingly affected by conditions such as infertility, polycystic ovary syndrome (PCOS), and endometriosis. Given the impact of modifiable lifestyle factors, nutrition has emerged as a potential preventive strategy. The Mediterranean Diet, characterised by high contents of anti-inflammatory and antioxidant compounds, may influence ovarian function, hormonal regulation, and reproductive outcomes. This study aimed to evaluate the association between adherence to the Mediterranean Diet and female reproductive health.

**Methodology:**

A systematic review was conducted according to PRISMA 2020 guidelines and registered in PROSPERO (CRD420261295562). PubMed, Scopus, ScienceDirect, and CINAHL databases were searched. Methodological quality was independently assessed using Joanna Briggs Institute critical appraisal tools. A meta-analysis of odds ratios was performed using the inverse variance method, and heterogeneity was assessed using Cochran’s *Q* and *I*^2^ statistics.

**Results:**

Fifteen unique studies were included in the systematic review and qualitative synthesis, of which two were eligible for quantitative meta-analysis. Higher dietary inflammatory potential, assessed by the Dietary Inflammatory Index (DII), was associated with an increased risk of female infertility (OR = 1.70; 95% CI: 1.23–2.34; *p* = 0.0013), with no observed heterogeneity (*I*^2^ = 0%). Qualitative evidence suggested that greater adherence to the Mediterranean Diet was associated with higher anti-Müllerian hormone levels, increased antral follicle counts, reduced risk of poor ovarian response (OR = 0.29), lower likelihood of endometriosis (OR = 0.06), fewer premenstrual symptoms, and improved blastocyst formation rates in assisted reproduction.

**Discussion:**

These findings support the potential role of anti-inflammatory dietary patterns in female reproductive health. Mediterranean Diet components, including omega-3 fatty acids, polyphenols, and monounsaturated fats, may reduce oxidative stress, improve insulin sensitivity, and modulate endocrine pathways. However, the certainty of evidence was rated as very low due to the predominance of observational studies.

**Conclusion:**

Observational evidence suggests that lower dietary inflammatory potential and greater adherence to the Mediterranean Diet are inversely associated with female infertility and adverse reproductive markers. However, because current evidence is overwhelmingly observational, these findings represent clinical associations rather than proven causal or therapeutic effects, highlighting the need for randomized controlled trials before clinical implementation.

**Systematic review registration:**

https://www.crd.york.ac.uk/prospero/display_record.php?ID=CRD420261295562, CRD420261295562.

## Introduction

Female reproductive health is a fundamental component of overall wellbeing in women of reproductive age and represents a major global public health challenge. In recent years, the prevalence of reproductive conditions has increased worldwide, including infertility, defined as the inability to achieve pregnancy after 12 months of regular unprotected sexual intercourse (17.5%) (1), polycystic ovary syndrome (3.4%) (2), endometriosis (10%) (3), and menstrual cycle disorders. These conditions have a substantial impact on quality of life, as well as on mental, physical, and social wellbeing. Female infertility, in particular, affects a large and increasing proportion of the population, placing a considerable burden on healthcare systems and public health resources (1).

Although reproductive function is influenced by biological and genetic factors, increasing evidence suggests a role for modifiable lifestyle factors, including body mass index, physical activity, obesity, stress, and diet (4). Within this context, nutrition, and specifically dietary patterns, has emerged as a relevant preventive and therapeutic factor, generating growing interest in the evaluation of dietary patterns with potential to improve female reproductive health (1). Studies conducted in women of reproductive age suggest that adherence to a balanced diet of high nutritional quality is associated with a lower likelihood of infertility and with improved reproductive outcomes (1).

The Mediterranean Diet is widely recognised as one of the healthiest dietary patterns, characterised by high consumption of fruits, vegetables, legumes, whole grains, nuts, fish, and olive oil as the principal source of fat, alongside moderate intake of dairy products, and lower consumption of red meat and ultra-processed foods (4). This dietary pattern has been associated with beneficial effects across cardiovascular, metabolic, inflammatory, and reproductive domains, largely attributed to its content of fibre, antioxidants, monounsaturated fatty acids, and bioactive compounds with anti-inflammatory properties (5).

In recent decades, research has examined the association between adherence to the Mediterranean Diet and reproductive health. Greater adherence has been associated with a lower prevalence of infertility, improved menstrual cycle regularity, and reduced symptom burden in conditions such as polycystic ovary syndrome and endometriosis (6). In addition, some studies suggest that women adhering to Mediterranean or similar dietary patterns may have a higher likelihood of conception compared with those following diets of lower nutritional quality (1, 4, 6–8). However, previous research presents conflicting findings regarding whether these benefits stem from the overall dietary matrix of the Mediterranean pattern or strictly from its low-inflammatory properties, creating a significant gap in knowledge. Furthermore, expected sources of clinical and methodological heterogeneity, such as varying study populations, clinical vs. general settings, and diverse dietary assessment tools, remain poorly explored.

From a pathophysiological perspective, several biological mechanisms may underlie these associations. The Mediterranean Diet may contribute to reduced chronic inflammation and oxidative stress, both of which are involved in ovarian and endometrial dysfunction (7). Its effects on hormonal regulation, insulin sensitivity, and gut microbiota composition may also be relevant to female reproductive function (5). Current evidence suggests that this dietary pattern may be associated with favourable changes in endometrial receptivity and metabolic balance, which may support implantation and embryonic development (9).

Despite increasing research interest, the available evidence remains heterogeneous, with conflicting findings regarding clinical outcomes. For instance, while some studies report clear benefits on clinical pregnancy rates, others fail to observe significant improvements (10). This heterogeneity is likely driven by the diverse definitions of dietary exposures and the variability in reproductive outcomes measured. Such discrepancies limit the ability to draw firm conclusions and highlight the critical knowledge gap that this systematic review aims to address.

Given the increasing interest in the role of dietary patterns in female reproductive health, a rigorous evaluation distinguishing between specific qualitative dietary habits and quantitative systemic inflammatory load is required. To address this, we explicitly define the two exposure variables under investigation: first, adherence to the Mediterranean Diet, which represents a qualitative dietary pattern rich in antioxidants, fiber, and monounsaturated fatty acids; and second, the Dietary Inflammatory Index (DII), a validated quantitative scoring tool designed to estimate the overall inflammatory potential of an individual’s diet. The primary reproductive outcomes of interest include ovarian reserve (objectively measured via Anti-Müllerian Hormone (AMH) and Antral Follicle Count (AFC)) and clinical female infertility (defined as the inability to achieve clinical pregnancy after 12 months of regular unprotected intercourse). Secondary outcomes encompass inflammatory-based gynaecological disorders such as Polycystic Ovary Syndrome (PCOS) and endometriosis.

This study addresses a major gap in the current literature: the historical failure to clearly differentiate between qualitative dietary quality metrics and quantitative inflammatory scores when analyzing reproductive health, which has led to conflicting findings regarding whether reproductive benefits stem from the diet’s matrix or its anti-inflammatory properties. Accordingly, the objective of this study is to systematically synthesize current qualitative evidence on the influence of the Mediterranean Diet on female reproductive health, while performing a quantitative meta-analysis specifically evaluating the association between dietary inflammatory potential (via DII) and female infertility. This review synthesizes both observational and interventional data, with the quantitative pooling strictly restricted to observational cohorts to ensure methodological homogeneity.

## Materials and methods

### Search strategy

A systematic bibliographic search was conducted in accordance with the PRISMA 2020 Statement (11), *Preferred Reporting Items for Systematic Reviews and Meta-Analyses*, a 27-item checklist addressing key aspects of systematic review reporting (12). The PubMed, Scopus, ScienceDirect, and CINAHL databases were searched. The review protocol was registered in the International Prospective Register of Systematic Reviews (PROSPERO) under the identifier CRD420261295562.

The search strategy was developed based on a structured research question defined using the PICO framework (13), specifying the population, exposure, comparator, and outcomes (Table 1).

**Table 1:** PICO format.

PICO component	Description
Population	Women of reproductive age, strictly defined as those aged 15–50 years, with no baseline fertility restrictions. This included cohorts with preserved reproductive function and populations with previous clinical diagnoses of subfertility or associated gynaecological endocrinopathies.
Exposure	High adherence to the Mediterranean Diet (assessed via standardized tools such as MEDAS, MDS, or MEDI-Lite) or lower dietary inflammatory potential / higher diet quality (assessed via validated indices including DII, E-DII, DQI-I, or DI-GM).
Comparator	Low adherence to the Mediterranean Diet, exposure to Western dietary patterns, or higher dietary inflammatory potential (operationalized through elevated scores on the Dietary Inflammatory Index [DII/E-DII] or lower scores on overall diet quality indices).
Outcomes	Primary outcomes: assessment of ovarian function and ovarian reserve using objective biomarkers, including serum anti-Müllerian hormone (AMH) concentrations and antral follicle count (AFC), together with indicators of clinical success in assisted reproductive techniques (ART), such as fertilisation rate, embryo development quality, blastocyst formation rate, implantation rate and clinical pregnancy rate. Secondary outcomes: prevalence, incidence or severity of clinical manifestations associated with the main gynaecological disorders with an inflammatory and metabolic basis, including polycystic ovary syndrome (PCOS), endometriosis and premenstrual syndrome (PMS).
Research question	What is the association between qualitative adherence to the Mediterranean Diet or quantitative dietary inflammatory potential (evaluated via DII/E-DII) and female reproductive health outcomes (including ovarian function, endocrine balance, gynaecological conditions, and clinical infertility) in women of reproductive age?

From this formulation, a search strategy was developed (Table 2), combining descriptors and keywords using the Boolean operators AND and OR to ensure precise linking of related terms. A comprehensive, systematic and reproducible literature search was conducted in the international biomedical databases and platforms PubMed (MEDLINE), Scopus, ScienceDirect and CINAHL, covering records published from January 2021 to March 2026. To maximise retrieval sensitivity for relevant articles, an advanced search strategy was developed by combining controlled vocabulary terms, including MeSH (Medical Subject Headings) terms where applicable, with free-text keywords searched in the title and abstract fields. Search terms were combined using the Boolean operators AND and OR, and the full search strategy was specified to ensure reproducibility.

**Table 2:** Search strategy.

Databases	Search strategy	Records found
PubMed	(“Mediterranean Diet” OR “Mediterranean dietary pattern” OR “Dietary Inflammatory Index” OR “DII” OR “E-DII”) AND (“female fertility” OR “infertility” OR “reproductive health” OR “ovarian function” OR “ovarian reserve” OR “endometriosis” OR “polycystic ovary syndrome”)	154
Scopus	(“Mediterranean Diet” OR “Mediterranean dietary pattern” OR “Dietary Inflammatory Index” OR “DII” OR “E-DII”) AND (“female fertility” OR “infertility” OR “reproductive health” OR “ovarian function” OR “ovarian reserve” OR “endometriosis” OR “polycystic ovary syndrome”)	312
Science direct	(“Mediterranean Diet” OR “Mediterranean dietary pattern” OR “Dietary Inflammatory Index” OR “DII” OR “E-DII”) AND (“female fertility” OR “infertility” OR “reproductive health” OR “ovarian function” OR “ovarian reserve” OR “endometriosis” OR “polycystic ovary syndrome”)	465
CINAHL	(“Mediterranean Diet” OR “Mediterranean dietary pattern” OR “Dietary Inflammatory Index” OR “DII” OR “E-DII”) AND (“female fertility” OR “infertility” OR “reproductive health” OR “ovarian function” OR “ovarian reserve” OR “endometriosis” OR “polycystic ovary syndrome”)	45
Search date: 25 March 2026	TOTAL	976

Following application of these operators, a targeted approach was incorporated to identify studies specifically addressing adherence to the Mediterranean Diet and its association with fertility and other female reproductive health parameters. This ensured that the search was both sensitive and sufficiently specific, focusing on studies that explicitly evaluated this relationship.

The final strategy was adapted for each database and explicitly incorporated terms reflecting both exposure variables (qualitative dietary patterns and quantitative inflammatory load) and reproductive outcomes. Search terms included “Mediterranean Diet,” “Mediterranean dietary pattern,” “Dietary Inflammatory Index,” “DII,” “E-DII,” “fertility,” “infertility,” “ovarian function,” “reproductive health,” “*in vitro* fertilisation,” “anti-Müllerian hormone,” “ovarian reserve,” and “endometriosis.” These terms were combined using AND to define relationships between concepts and OR to expand variations within each concept, ensuring that studies evaluating both adherence to the Mediterranean Diet and dietary inflammatory potential were systematically retrieved.

### Inclusion criteria

Studies were included if they involved women of reproductive age (15–50 years); assessed qualitative adherence to the Mediterranean Diet (e.g., via MEDAS, MDS, MEDI-Lite) or quantitative dietary inflammatory potential and diet quality metrics (e.g., via DII, E-DII, DQI-I, or DI-GM); examined associations with indicators of female reproductive health, including ovarian function, menstrual cycle characteristics, oocyte quality, fertility, or outcomes in assisted reproductive techniques; and used primary quantitative, analytical, epidemiological, or empirical designs, including randomized controlled trials (RCTs), prospective or retrospective cohort studies, appropriately matched case–control studies, and analytical cross-sectional studies. This specific time frame was strategically selected to provide an update to previous, well-established comprehensive systematic reviews that have extensively analysed the pre-2021 evidence regarding the Mediterranean Diet and female reproduction. Rather than duplicating these previous efforts, this review focuses on the most recent body of evidence emerging from the post-COVID-19 dietary landscape and the contemporary integration of specific inflammatory metrics, such as the Dietary Inflammatory Index (DII). By doing so, we aim to provide a targeted synthesis of the latest epidemiological trends and newly available data in this rapidly evolving field.

This time frame was selected to capture recent, methodologically robust evidence reflecting contemporary clinical practice, health behaviours, and diagnostic criteria following the COVID-19 pandemic. Language restrictions were applied to ensure accuracy in data extraction and to minimise misinterpretation of complex clinical and nutritional terminology.

### Exclusion criteria

Studies were excluded if they:

- Included postmenopausal women, male populations, or animal models.
- Evaluated dietary patterns or nutritional metrics unrelated to either the Mediterranean Diet or dietary inflammatory potential (e.g., isolated nutrient supplements or single-food interventions lacking standardized dietary quality or inflammatory indices).
- Were secondary literature, including narrative reviews, systematic reviews, meta-analyses, consensus statements, and editorial letters.
- Were duplicate records across databases.
- Were published in languages other than English or Spanish.
- Did not provide sex-disaggregated data.
- Were secondary literature, including narrative reviews, letters to the editor, editorial opinions, consensus statements, commentaries, clinical guidelines, systematic reviews or previous meta-analyses.
- Presented major methodological shortcomings in the reporting of variables, incomplete data, or insufficient sex-disaggregated data or measures of dispersion to allow extraction or calculation of odds ratios (ORs) and their corresponding 95% confidence intervals (95% CIs) required for the quantitative synthesis.

### Delimitation of the variables analysed and selection criteria justifying the inclusion of both Mediterranean Diet and DII studies

To avoid conceptual inconsistencies in the variables analysed, the selection strategy clearly differentiated between Mediterranean diet adherence metrics, such as MEDAS or MDS, and dietary inflammatory burden indices, such as DII or E-DII. Methodologically, the meta-analysis was restricted to studies sharing a homogeneous definition of both the exposure variable, DII, and the primary outcome, infertility, thereby supporting the internal validity of the quantitative model. The remaining phenotypic variables, including ovarian reserve and menstrual symptoms, were evaluated through qualitative synthesis.

### Analytical methods

For quantitative synthesis, StatsDirect software was used. A meta-analysis of odds ratios (OR) was performed using the inverse variance method. It is imperative to clarify that this meta-analysis exclusively synthesizes data from observational studies. Clinical trial data were strictly restricted to the qualitative synthesis to prevent the methodological bias of comparing fundamentally different effect sizes. Fully adjusted multivariable OR estimates and their corresponding 95% confidence intervals were extracted from primary studies, ensuring that extracted effect sizes are controlled for major clinical confounders (such as age, BMI, and energy intake). To ensure methodological comparability across studies, the quantitative meta-analysis specifically pooled estimates comparing the highest vs. lowest category of dietary inflammatory potential (DII score). Studies evaluating energy-adjusted DII (E-DII) or self-reported fertility issues rather than clinically diagnosed infertility (such as Simon-Alesi et al.) were intentionally excluded from quantitative pooling to avoid mixing disparate exposure metrics and outcome definitions and were instead synthesised narratively.

Statistical heterogeneity was assessed using Cochran’s *Q* statistic, the *I*^2^ index, and between-study variance estimated using the DerSimonian-Laird method. To ensure the robustness of the synthesis, both fixed-effects and random-effects models were calculated, and consistency of the pooled estimates was compared across approaches.

Publication bias was assessed using the Begg-Mazumdar test (Kendall’s rank correlation) and Egger’s linear regression. However, the interpretability of these tests was considered limited due to the small number of included studies, which constrains the assessment of funnel plot asymmetry.

Importantly, interventional data (such as clinical trials) and qualitative studies evaluating Mediterranean diet scores (e.g., MEDAS) were excluded from quantitative synthesis. No data from clinical trials were pooled with observational cohort or case–control findings. The meta-analysis was strictly restricted to primary observational studies reporting adjusted odds ratios (OR) for the association between the Dietary Inflammatory Index (DII) and infertility, preventing the mixing of disparate effect sizes.

### Assessment of methodological quality

The selection phase was conducted independently by two researchers. Discrepancies identified during screening were resolved through consultation with a third researcher.

Methodological quality was assessed independently by two reviewers using the critical appraisal instruments of the Joanna Briggs Institute, University of Adelaide. Additionally, the certainty of the evidence for the meta-analysed outcomes was assessed using the Grading of Recommendations Assessment, Development and Evaluation (GRADE) approach. Given the observational nature of the included primary studies, the initial certainty of the evidence was rated as low and then assessed across the GRADE domains of risk of bias, inconsistency, indirectness, imprecision, and publication bias. Methodological quality was assessed using the specific Joanna Briggs Institute (JBI) critical appraisal tools adapted to each study design, utilizing the 8-item checklist for cross-sectional studies, the 10-item checklist for case–control studies, 13- items checklist for clinical trials, and the 11-item checklist for cohort studies. Methodological quality scores ranged from 8/8 to 11/11, depending on the specific instrument.

## Results

The identification and selection of studies followed the PRISMA 2020 Statement, ensuring transparency and methodological rigour. A total of 976 records were retrieved from Scopus, PubMed, ScienceDirect, and CINAHL. After removal of duplicate records (*n* = 496), 480 unique records were retained for screening.

**Figure 1:**
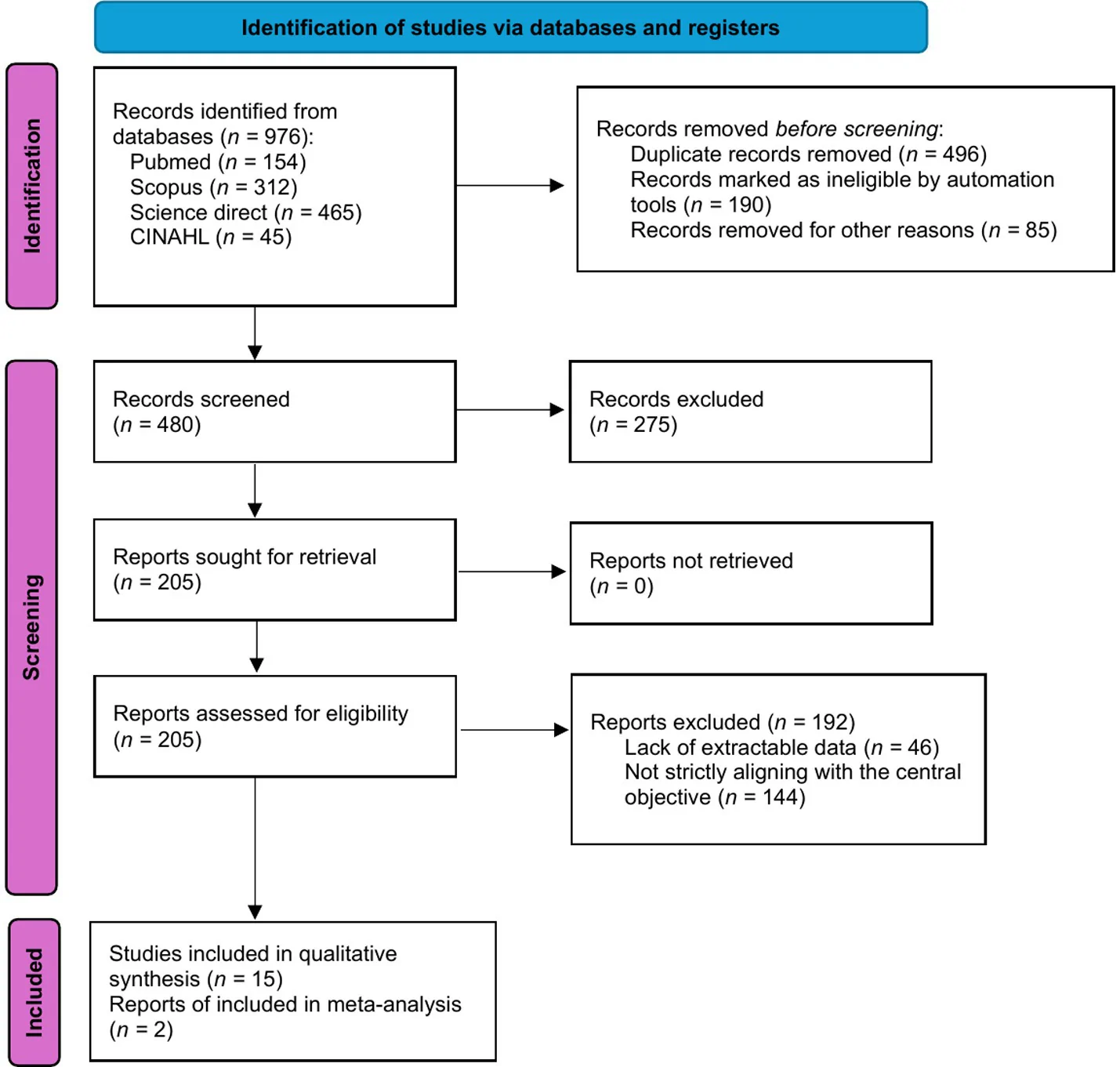
Search results (PRISMA flow chart).

During title and abstract screening, 275 records were excluded for not meeting the primary eligibility criteria: 190 due to publication outside the five-year time frame, 71 due to language restrictions, and 14 for not reporting sex-disaggregated data (Figure 1).

This process resulted in the preselection of 205 articles for full-text assessment. During the eligibility phase, 192 articles were excluded due to insufficient data or methodological limitations (*n* = 48), or lack of alignment with the study objective following full-text review (*n* = 144). Following this critical appraisal and the application of the definitive inclusion criteria, 15 unique studies were included in the systematic review and qualitative synthesis, of which 2 were included in the quantitative meta-analysis.

The methodological characteristics of the included studies (*n* = 15) were as follows: 5 cross-sectional studies, 2 descriptive cross-sectional studies, 5 case–control studies, 2 prospective cohort studies, and 1 clinical trial.

In terms of geographical distribution, the included studies showed a predominantly European contribution, led by Spain (*n* = 3) and Italy (*n* = 3), followed by a geographically diverse representation, with one study each from Iran, Australia, China, Palestine, Turkey, and Ethiopia (Table 3).

**Table 3:** Characteristics of the studies included in the systematic review.

Study	Country	Study design	Quality assessment	Main findings
Molina et al. (7)	Spain	Analytical cross-sectional	8/8	The MD is associated with a healthier endometrial metabolomic profile, characterised by modulation of lipid and amino acid pathways, and with lower levels of inflammation and improved uterine function, particularly in women without underlying dysfunction.
Noli et al. (14)	Italy	Analytical cross-sectional	8/8	Unexpected poor ovarian response (≤3 oocytes despite preserved ovarian reserve) was less likely in women in the second tertile of the MDS compared with the first tertile (adjusted OR = 0.29; 95% CI 0.11–0.76), supporting a protective effect of this pattern on ovarian stimulation.
Rahele-Ziaei et al. (15)	Iran	Case–control	10/10	Higher adherence to DQI-I was associated with higher antral follicle count in women with diminished ovarian reserve. After adjusting for potential confounders, the odds of diminished ovarian reserve decreased with increasing DQI-I score (OR = 0.39; 95% CI 0.18–0.86).
Almahareeq et al. (2)	Palestine	Case–control	9/10	PCOS was associated with greater severity of PMS symptoms and poorer mental health outcomes. Lower adherence to the Mediterranean Diet was also observed. Regression analysis showed associations with higher GHQ scores (OR = 1.09; 95% CI 1.03–1.16), lower MD adherence (OR = 0.86; 95% CI 0.76–0.98), and increased PMS severity, particularly physical symptoms.
Noormohammadi et al. (16)	Iran	Case–control	9/10	High adherence to the Mediterranean Diet (MEDI-Lite score > mean) was associated with a 94% reduction in the odds of endometriosis (OR = 0.06; 95% CI 0.02–0.17; *p* < 0.001). Similarly, women with higher HDI scores had 95% lower odds of endometriosis (adjusted OR = 0.05; 95% CI 0.02–0.12; p < 0.001). These findings suggest an inverse association between endometriosis and adherence to both the Mediterranean Diet and HDI.
Zhang et al. (5)	China	Cross-sectional	7/8	Lower DI-GM scores were associated with a higher prevalence of infertility, suggesting a role of diet quality linked to gut microbiota.
Martín-Manchado et al. (1)	Spain	Case–control	9/10	Infertile women showed significantly lower muscle mass (*p* = 0.005) and larger hip circumference (*p* = 0.034). High red meat consumption was associated an increased risk of female infertility (*p* = 0.011).
Martín-Manchado et al. (17)	Spain	Descriptive cross-sectional	8/8	Ovarian reserve was assessed using AMH (mean 2.32 ± 1.59 ng/mL) and AFC (mean 19.80 ± 14.13). A statistically significant association was observed between low adherence to the Mediterranean Diet and lower AMH levels (*p* = 0.025). Additionally, low vegetable intake (*p* = 0.044), high red meat consumption (*p* = 0.027), and carbonated beverage intake (*p* = 0.015) were associated with lower AMH levels, indicative of reduced ovarian reserve. Low fruit intake was also associated with lower oestradiol levels (*p* = 0.045).
Simon-Alesi et al. (4)	Australia	Cross-sectional	7/8	A diet with greater inflammatory potential was associated with higher odds of self-reported fertility problems (adjusted odds ratio [aOR] per 1-unit increase in E-DII = 1.13; 95% CI 1.06–1.19), with significant differences between the highest and lowest E-DII quartiles (aOR = 1.53; 95% CI 1.23–1.90). Higher dietary quality was associated with lower odds of self-reported fertility problems (aOR per 1-unit increase in DGI = 0.99; 95% CI 0.99–0.99), including when comparing highest and lowest DGI quartiles (aOR = 0.76; 95% CI 0.61–0.95).
Wakwoya et al. (21)	Ethiopia	Clinical trial	10/13	The intervention package provided three counselling sessions by trained midwives, three-page take-home brochures prepared in local languages, and the delivery of 18 weekly serial short text messages. The women in the control group received routine nutrition education. The mean mid-upper arm circumference in the intervention group increased by 1.8% (23.08 vs. 23.44, *p* < 0.01). Similarly, the proportion of undernutrition in the intervention group was 11% (25 vs. 36%, *p* = 0.02) lower compared to the control arm. At the end of the trial, women in the intervention arm had significantly better nutritional status than women in the control group (*β* = 0.47, *p* < 0.01).
Tuğçe-Odabaş (22)	Turkey	Cross-sectional	8/8	85 of the 207 participating pregnant women (41.1%) were diagnosed with GDM. According to Logistic Regression models, age (OR = 1.088; 95% CI 1.031–1.149) and infertility treatment (OR = 4.570; 95% CI 1.443–14.474) significantly increased the occurrence of GDM, while adherence to the MD (OR = 0.683; 95% CI 0.568–0.820) was associated with reduced risk.
Huijun-Chen (20)	China	Prospective cohort	11/11	517 participants followed low to moderate MeDiet, and only a small group of them (*n* = 88) followed high MeDiet. High adherence to the Mediterranean Diet (MeDiet score 8–14) was associated with higher blastocyst formation rates (46.08%) compared with low (41.75%) and moderate adherence groups (40.07%; p = 0.044).
Liprino (18)	Italy	Prospective cohort	10/11	The mean MEDAS score was 7.6 ± 1.2: 93% of women showed moderate adherence, 3% high adherence, and 4% low adherence. No significant associations were found between MEDAS score, and total oocytes retrieved, MII oocytes, or clinical pregnancy. The findings underscore the need for structured nutritional counselling to reinforce sustained adherence and support long-term reproductive health.
Wang et al. (23)	China	Cross-sectional	8/8	Higher DII scores were significantly associated with an increased risk of female infertility (adjusted OR = 1.61; 95% CI 1.12–2.31)
KabodMehri et al. (8)	Iraán	Case–control	9/10	Higher DII scores were significantly associated with increased odds of female infertility (adjusted OR = 2.08; 95% CI 1.02–4.25)

AMH, Anti-Müllerian hormone; aOR, Adjusted odds ratio; ART, Assisted reproductive technology; DGI, Diet Quality Index; DII, Dietary inflammatory index; DI-GM, Gut microbiota dietary index; MD, Mediterranean Diet; GDM, Gestational diabetes mellitus; DOR, Diminished ovarian reserve; DQI-I, Diet Quality Index-International; E-DII, Energy-adjusted Dietary Inflammatory Index; HDI, Healthy Diet Indicator; 95% CI, 95% confidence interval; BMI, Body mass index; MDS, Mediterranean Diet Score; MEDAS, Mediterranean Diet Adherence Screener; MII, Metaphase II oocytes (mature oocytes); MUAC, Mid-upper arm circumference; OR, Odds ratio; WHR, Waist-to-hip ratio; AFC, Antral follicle count; OvR, Ovarian reserve; PCOS, Polycystic ovary syndrome; PMS, Premenstrual syndrome.

### Quantitative synthesis

Variables associated with ovarian reserve, such as anti-Müllerian hormone levels and antral follicle count, were not statistically pooled or subjected to meta-analysis because of the marked methodological diversity in the designs of the primary studies, including Noli et al. and Rahele-Ziaei et al. (14, 15). The individual estimates reported in these studies, which suggested a lower likelihood of impaired ovarian function, with ORs ranging from 0.29 to 0.39, were therefore addressed exclusively in the qualitative synthesis.

The only formal meta-analysis conducted in this review examined the association between the Dietary Inflammatory Index (DII) and infertility. The pooled analysis showed a statistically significant association between higher dietary inflammatory potential and female infertility, with a pooled effect estimate of OR = 1.70 (95% CI: 1.23–2.34; *p* = 0.0013; Z = 3.21), indicating that women with higher DII scores have a 70% greater likelihood of infertility compared with those following more anti-inflammatory dietary patterns. However, as the quantitative synthesis was based on only two primary studies, the results should be interpreted with caution, and the overall robustness of the model remains limited by the small body of available evidence.

**Figure 2:**
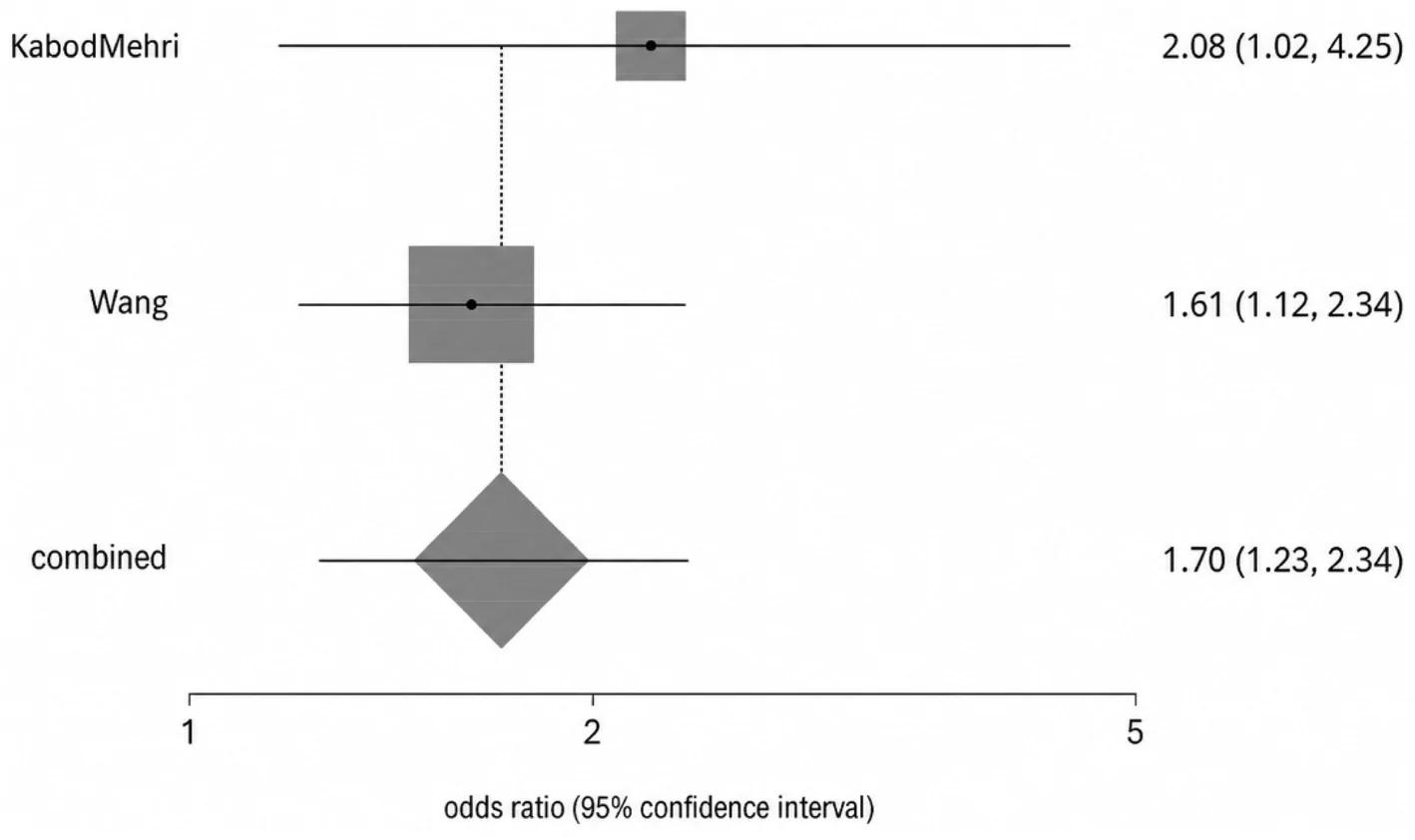
Forest plot of the meta-analysis (fixed-effect model).

This quantitative synthesis specifically pooled fully adjusted odds ratios representing the highest vs. lowest DII score contrast for clinically diagnosed female infertility. The study by Wang et al. (16) provided the fully adjusted multivariable estimate comparing extreme DII score quantiles (adjusted OR = 1.61; 95% CI: 1.12–2.31), contributing the largest statistical weight to the model. Similarly, KabodMehri et al. (8) evaluated extreme DII tertiles, reporting a consistent fully adjusted effect size (adjusted OR = 2.08; 95% CI: 1.02–4.25). Both studies employed fully adjusted models controlling for key potential confounders, supporting the comparability of the pooled contrast (Figure 2).

**Figure 3:**
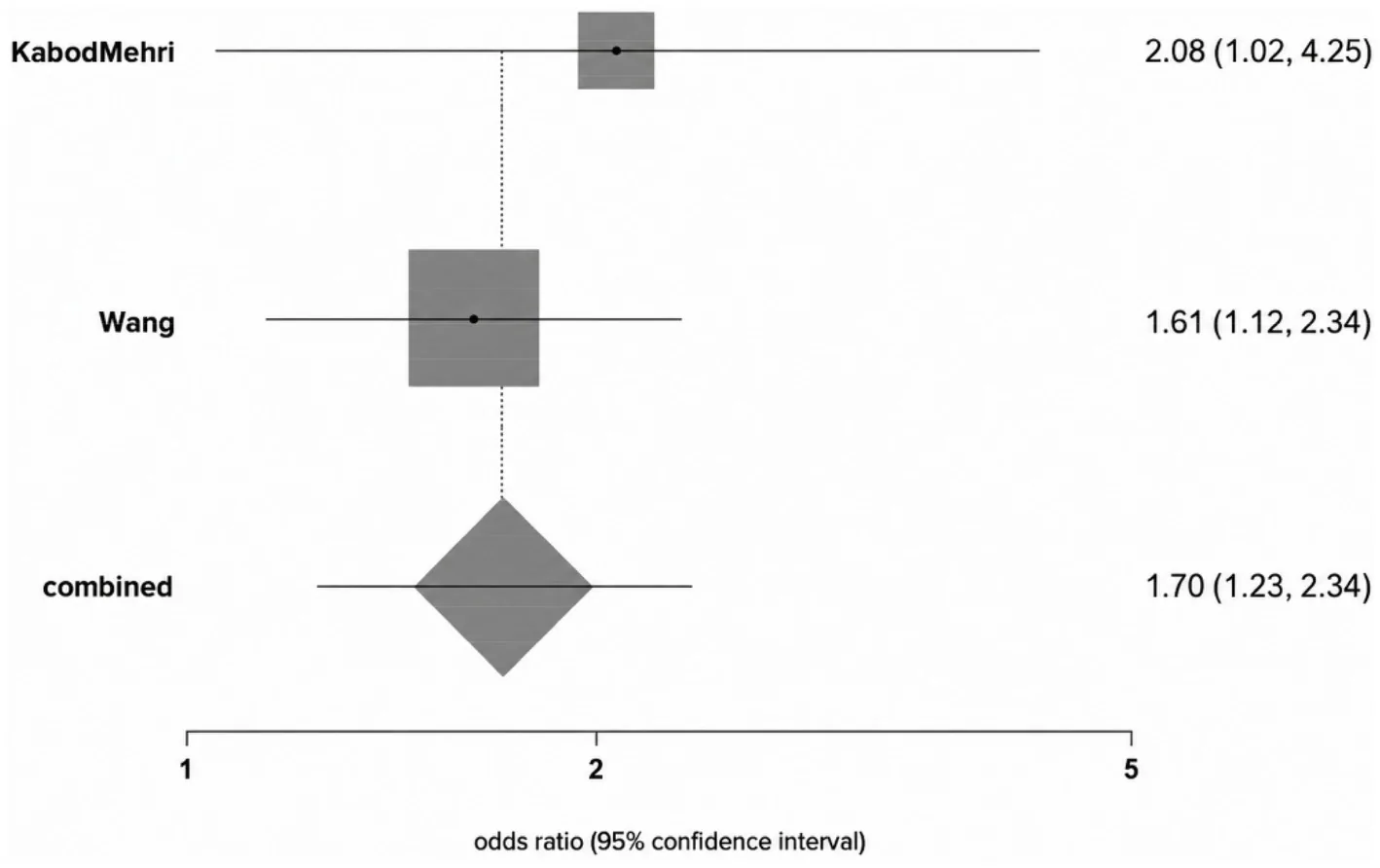
Forest plot of the meta-analysis (random effect).

Heterogeneity analysis revealed no evidence of between-study heterogeneity (Cochran’s *Q*, *p* = 0.5294). Consistent with this, the between-study variance was estimated as zero, and the fixed-effects and random-effects models yielded identical results. While this lack of statistical heterogeneity is encouraging, it does not inherently guarantee the internal validity or generalizability of the model, as the statistical power of the Cochran’s *Q* and *I*^2^ tests is critically limited when evaluating only two studies (Figure 3).

### Methodological limitations

It should be noted that, owing to the limited number of studies included (*n* = 2), it was not possible to perform an informative assessment of publication bias using the Begg–Mazumdar and Egger tests. Nevertheless, the direction of the effect was consistent across both studies, suggesting a positive dose–response relationship between the inflammatory potential of the diet and the risk of infertility (Figure 3).

### Traceability and scientific coherence matrix

The following table outlines the correspondence between the revised objective, the included studies according to their variables, and the synthesis method applied (Table 4).

**Table 4:** Traceability and scientific coherence matrix.

Exposure variable	Outcome variables	Included studies	Type of synthesis applied	Synthesised finding
Dietary Inflammatory Index (DII or E-DII)	Clinically diagnosed or self-reported female infertility	Wang et al. (23), KabodMehri et al. (8), Simon-Alesi et al. (4)	Quantitative meta-analysis for Wang and KabodMehri; qualitative synthesis for Simon-Alesi	Pooled OR = 1.70 (95% CI: 1.23–2.34). Higher dietary inflammatory potential was associated with a 70% higher likelihood of infertility.
Adherence to the Mediterranean diet (MDS, MEDAS, MEDI-Lite)	Ovarian reserve, including anti-Müllerian hormone (AMH), antral follicle count (AFC), and response to ovarian stimulation	Noli et al. (14), Martín-Manchado et al. (17), Rahele-Ziaei et al. (15)	Qualitative synthesis, owing to marked heterogeneity in methodological criteria	Significant positive association between high adherence and preservation of ovarian function, with a lower likelihood of poor ovarian response.
Adherence to the Mediterranean diet or overall diet quality	Gynaecological conditions with an endocrine or inflammatory basis, including PCOS, endometriosis, and premenstrual syndrome	Almahareeq et al. (2), Noormohammadi et al. (16)	Qualitative synthesis	Reduction of up to 94% in the likelihood of endometriosis, together with a more favourable metabolic and endocrine profile in patients with PCOS.
Adherence to the Mediterranean diet	Assisted reproductive technology (ART) outcomes, including embryo quality and blastocyst formation rate	Huijun-Chen (20), Liprino (18), Molina et al. (7)	Qualitative synthesis	Higher embryo development and blastocyst formation rates (46.08%), together with a more favourable endometrial metabolic profile for implantation.

While the qualitative synthesis suggests a potential benefit of the Mediterranean Diet on female reproductive capacity, these findings must be critically interpreted due to marked clinical and methodological heterogeneity across the included studies. The evidence does not constitute a uniform body; rather, it encompasses highly diverse populations and evaluates distinct pathophysiological outcomes, ranging from clinical infertility and ovarian reserve to PCOS, endometriosis, and assisted reproduction parameters. Furthermore, the reliance on varied dietary assessment methods (e.g., MEDAS, MDS, DQI-I) and differing outcome definitions precludes a homogenous interpretation. Consequently, the observed findings should be viewed as context-specific associations rather than a uniform protective effect, highlighting the need to approach this evidence with methodological caution.

There is growing evidence that the Mediterranean Diet acts as a modulator of the hypothalamic–pituitary-ovarian axis, supporting endocrine homeostasis (9). This effect is particularly robust in women with polycystic ovary syndrome, where the dietary pattern contributes to improvements in insulin resistance and hyperandrogenism. The case–control study by Almahareeq et al. showed that low adherence to the Mediterranean pattern was associated with a higher prevalence of polycystic ovary syndrome, even after adjustment for age, body mass index, and smoking status. These findings point to the role of the Mediterranean pattern as a tool for hormonal modulation in women of reproductive age (2).

Likewise, the Mediterranean Diet appears to exert a protective effect against endometriosis, a condition closely linked to chronic pelvic pain and inflammation. Noormohammadi et al. (16) reported that high adherence to this dietary pattern was associated with up to a 94% reduction in the likelihood of endometriosis (OR = 0.06; 95% CI 0.02–0.17). This protective effect was linked to higher consumption of fruits, vegetables, legumes, fish, and olive oil, as opposed to pro-inflammatory dietary patterns characterised by high intake of red meat and ultra-processed foods.

Consistent with this, Martín-Manchado et al. (17) reported that greater consumption of pro-inflammatory foods, including carbonated beverages and red meat, was associated with poorer reproductive health markers and reduced ovarian reserve. In addition, lower adherence to the Mediterranean Diet has been associated with greater severity of premenstrual syndrome symptoms, as reported by Almahareeq et al. (2).

Mechanistically, these findings may be explained by the anti-inflammatory and antioxidant properties of the Mediterranean Diet, including polyphenols and omega-3 fatty acids, which act on pathophysiological pathways involved in uterine inflammation and pain. In line with this, Molina et al. (7) reported that adherence to this dietary pattern was associated with a healthier endometrial metabolomic profile, contributing to reduced inflammation and improved uterine function. Overall, the evidence supports the role of anti-inflammatory dietary patterns in modulating processes associated with menstrual pain and reproductive wellbeing.

With regard to reproductive outcomes, adherence to the Mediterranean dietary pattern has been associated with a lower risk of infertility and a higher likelihood of conception in population-based and cohort studies like the ones by Martín-Manchado et al. and Alesi et al. (1, 4). In line with this, Liprino et al. emphasised the importance of maintaining dietary adherence before, during, and after pregnancy, given its relevance across the reproductive continuum (18). In assisted reproduction, the meta-analysis by Dong et al. reported higher clinical pregnancy rates and improved embryo implantation among women adhering to the Mediterranean Diet, although no significant differences were observed in live birth rates (19). In addition, Chen et al. reported a higher number of blastocysts, a key indicator of embryo quality, among women with greater adherence (20).

Building on the findings of Martín-Manchado et al. (17), emerging evidence suggests that adherence to high-quality dietary patterns, particularly the Mediterranean Diet, may directly influence oocyte competence through reduced oxidative stress and improved ovarian metabolic environment. Consistent with this, Noli et al. (14) reported that greater adherence significantly reduced the likelihood of poor ovarian response during *in vitro* fertilisation stimulation, indicating a potential impact on oocyte quality and maturation. Complementing these findings, the qualitative synthesis of this review suggested that higher dietary quality was associated with reduced impairment of ovarian function (with individual ORs ranging from 0.29 to 0.39), supporting the role of antioxidant and anti-inflammatory components, including polyphenols and monounsaturated fatty acids, in preserving ovarian reserve and promoting a more favourable follicular microenvironment (14, 15).

Adherence to the Mediterranean Diet also plays a key role in endocrine regulation and ovarian function. Martín-Manchado et al. (1, 17) demonstrated that higher dietary quality was associated with increased levels of anti-Müllerian hormone and greater antral follicle count, whereas pro-inflammatory dietary patterns were linked to reduced ovarian reserve. Nutritional interventions based on the Mediterranean pattern have also been associated with improvements in insulin sensitivity and the testosterone to sex hormone-binding globulin ratio, both of which are central to reproductive axis homeostasis (7). In parallel, dietary quality influences menstrual symptomatology, with lower adherence associated with greater severity of premenstrual syndrome symptoms (2). Overall, these findings support the role of dietary modulation of inflammatory processes as an effective strategy for improving female reproductive health.

Taken together, the evidence from this systematic review indicates that the Mediterranean Diet constitutes a non-pharmacological intervention with meaningful effects across multiple domains of female reproductive health, acting as a key modulator from the preconceptional stage through to the management of specific conditions. In women with polycystic ovary syndrome, adherence to this dietary pattern has been associated with improvements in hormonal and metabolic parameters that appear to be independent of caloric restriction (9). These benefits extend to assisted reproduction, where pre-treatment adherence has been linked to improved embryo quality and implantation rates (19, 20). Similarly, in inflammatory conditions such as endometriosis and dysmenorrhoea, adherence to the Mediterranean Diet has been associated with lower levels of pro-inflammatory markers and reduced symptom severity (6, 7, 16).

From a public health and preventive medicine perspective, the literature highlights the importance of structured nutritional education programmes. Wakwoya et al. (21) demonstrated that culturally adapted educational interventions improved nutritional status and reduced the prevalence of malnutrition in pregnant women. These findings support the integration of nutritional education into clinical practice as a strategy to reduce the risk of infertility, gestational diabetes, and obstetric complications (18, 21). From a public health and preventive medicine perspective, incorporating screening tools such as the MEDAS-14 questionnaire into routine clinical reproductive care remains premature given that almost all available evidence is observational and graded as very low certainty under the GRADE criteria. Large-scale, well-designed randomized controlled trials are strictly required to establish their definitive clinical utility before integration into primary care can be recommended (1).

The effectiveness of these interventions appears to be maximised when initiated during the preconception period, ideally at least 3 months prior to conception, allowing for the mitigation of systemic metabolic risk factors. In this context, Odabaş et al. (22) reported that higher adherence to the Mediterranean Diet acted as a protective factor against the incidence of gestational diabetes mellitus, potentially mediated by its beneficial effects on gut microbiota and metabolic regulation (20). With regard to menstrual health, the findings indicate an inverse association between dietary quality and physical symptomatology. Specifically, Almahareeq et al. (2) reported that lower adherence to antioxidant dietary patterns and higher consumption of ultra-processed foods were associated with greater severity of premenstrual syndrome symptoms, supporting the role of nutritional intervention not only as a preventive measure, but also as a relevant clinical management strategy in reproductive health.

## Discussion

### Main interpretation of the findings

The findings of this meta-analysis suggest a statistically significant association between the inflammatory potential of the diet and female infertility, although the robustness of this conclusion is limited by the inclusion of only two eligible studies. The pooled estimate (OR = 1.70; 95% CI: 1.23–2.34; *p* = 0.0013) suggests that women with more pro-inflammatory dietary patterns, reflected in higher Dietary Inflammatory Index (DII) scores, have a 70% higher likelihood of infertility than those with a lower dietary inflammatory potential.

A key aspect of these results is the absence of observed heterogeneity between the included studies (*I*^2^ = 0%; Cochran’s *Q*, *p* = 0.5294), which suggests consistency between the studies by KabodMehri et al. and Wang et al. (8, 23). The pooled estimate was primarily driven by Wang et al., which contributed approximately 80% of the total statistical weight, whereas KabodMehri et al. (8), despite being based on a smaller sample, reported a larger effect estimate (OR = 2.08), consistent with the overall direction of the association.

From a clinical perspective, these findings support a positive dose–response pattern, whereby greater inflammatory potential of the diet is associated with a higher likelihood of reproductive impairment. Although the limited number of studies (*n* = 2) restricts the formal assessment of publication bias using Egger’s or Begg’s tests, the consistency in both the direction and magnitude of the effect across studies supports the internal validity of the pooled estimate.

Collectively, these findings highlight the relevance of nutrition not only as a general health factor but also as a therapeutic approach in the management of infertility. Our quantitative meta-analysis suggests a potential benefit in reducing dietary inflammatory potential (DII) to improve reproductive outcomes; however, given the extremely limited quantitative evidence base, these clinical implications must be interpreted with strict caution. While our qualitative synthesis suggests the Mediterranean Diet is a clinically relevant anti-inflammatory pattern, it is crucial to note that our quantitative evidence evaluated the DII rather than Mediterranean Diet adherence per se.

### Biological plausibility: metabolic, endocrine, and inflammatory axes

While the Mediterranean Diet is theoretically associated with the modulation of key metabolic and endocrine pathways relevant to ovarian function and steroidogenesis (24) these pathways were not directly evaluated at a molecular level in the primary settings of the majority of the included studies. The clinical trial by Scannell et al. reported that a 12-week Mediterranean Diet intervention improved insulin sensitivity, lipid profile, and androgen balance, reflected in the testosterone to sex hormone-binding globulin ratio, supporting its biological plausibility in women with polycystic ovary syndrome (9). Complementarily, Almahareeq et al. showed that lower adherence to the Mediterranean Diet was associated with a higher prevalence of polycystic ovary syndrome, even after adjustment for age, body mass index, and smoking status (2). While these clinical associations suggest that the effects of the Mediterranean Diet might extend beyond simple weight control (5), the underlying biological mechanisms, such as oxidative stress reduction, systemic anti-inflammatory actions, and endometrial receptivity, remain largely hypothetical within the context of the reviewed literature. Consequently, these proposed pathways must be interpreted with caution due to the lack of direct physiological and laboratory measurements in the primary studies.

Although not directly measured in the primary studies included in this review, previous literature provides biological plausibility, suggesting that characteristic components of the Mediterranean dietary pattern (such as omega-3 fatty acids and polyphenols) may theoretically contribute to the modulation of inflammatory responses and oxidative stress (5, 7). In line with this, Martín-Manchado et al. and Liprino et al. reported higher levels of anti-Müllerian hormone and greater antral follicle count in women with higher adherence, both recognised indicators of ovarian reserve (17, 18), suggesting that these dietary components actively preserve reproductive reserve, a systemic benefit that likely extends beyond female-specific physiology. Furthermore, adherence to the Mediterranean diet must be conceptualized as a fundamental pillar of a healthy lifestyle that comprehensively influences overall reproductive function. This perspective is strongly supported by recent evidence evaluating dietary patterns and reproductive outcomes in broader contexts. Specifically, recent studies investigating male fertility (25, 26) provide valuable complementary evidence to this paradigm. Although these investigations focus on male reproductive outcomes, they reinforce the overarching concept that Mediterranean dietary patterns actively contribute to reproductive health across both sexes. This shared benefit is primarily mediated through systemic anti-inflammatory and antioxidant pathways, which mitigate oxidative stress and preserve cellular integrity in both ovarian and testicular microenvironments.

### Menstrual cycle: pain, inflammation, and quality of life

These findings are consistent with the anti-inflammatory effects of omega-3 fatty acids and polyphenols, including their role in gut microbiota and immunometabolic pathways. Symptomatic improvements may translate into indirect benefits in quality of life and reproductive wellbeing (5).

With regard to the menstrual cycle, the findings of this review suggest that the Mediterranean Diet may modulate inflammatory and immunometabolic processes involved in menstrual symptomatology (27). A higher intake of antioxidant and anti-inflammatory compounds, including polyphenols, monounsaturated fatty acids, and omega-3 fatty acids, appears to contribute to a less pro-inflammatory uterine environment. This interpretation is consistent with the findings of Molina et al. (7), who reported that greater adherence to this dietary pattern was associated with a more balanced endometrial metabolomic profile and reduced activation of inflammatory pathways, which may explain lower menstrual pain and improved endometrial function.

Conversely, lower dietary quality appears to aggravate cycle-related symptoms. Almahareeq et al. (2) observed that women with lower adherence to the Mediterranean Diet exhibited greater severity of the physical symptoms of premenstrual syndrome, suggesting an interaction between systemic inflammation, dietary patterns, and menstrual wellbeing. These physiological and clinical observations as a whole support the notion that dietary modulation may contribute to pain regulation, reduced inflammatory burden, and overall improvements in reproductive wellbeing (2, 7, 17).

### Fertility and reproductive outcomes (natural and assisted reproduction)

Most studies report a favourable association between adherence to the Mediterranean Diet and female fertility. In population-based settings, higher dietary quality has been linked to a lower likelihood of infertility and a greater probability of conception (1, 4). Martín-Manchado et al. reported that infertile women consumed more ultra-processed foods, whereas fertile women more frequently adhered to a Mediterranean dietary pattern (1, 17). Similarly, Alesi et al. found that greater adherence to the Mediterranean Diet was associated with a lower likelihood of infertility, even in the absence of direct measurements of follicle-stimulating hormone, luteinising hormone, or anti-Müllerian hormone, suggesting a potential indirect relationship with oocyte quality (4).

In assisted reproductive techniques, the meta-analysis by Dong et al. reported higher clinical pregnancy and implantation rates among women with greater adherence to the Mediterranean Diet, although no significant differences were observed in live birth rates (19). Chen et al. reported a higher proportion of blastocysts and improved indicators of embryo quality in women with higher adherence, findings consistent with a more favourable ovarian and endometrial environment (20). In contrast, Liprino et al. found no significant association between adherence to the Mediterranean Diet and clinical pregnancy rates or number of oocytes, which may reflect differences in sample size and dietary assessment instruments (MEDAS compared with food frequency questionnaires) (18).

### Ovarian reserve and oocyte quality: heterogeneity and potential sources

A critical limitation of the current literature is the high level of clinical and methodological heterogeneity across the evaluated studies. Research analyzing infertility, ovarian reserve, PCOS, endometriosis, and menstrual outcomes is often discussed in tandem, despite representing fundamentally different clinical populations, distinct dietary assessment metrics (e.g., MEDAS, MDS, DQI-I), and highly variable outcome definitions. This diversity precludes a uniform clinical interpretation and highlights that the qualitative benefits of the Mediterranean diet cannot be generalized as a homogeneous protective effect across all reproductive phenotypes.

Findings relating to ovarian reserve and oocyte quality show considerable variability. Martín-Manchado et al. reported that higher adherence to the Mediterranean Diet was associated with increased levels of anti-Müllerian hormone and greater antral follicle count, whereas Liprino et al. did not observe significant associations with assisted reproduction outcomes (17, 18). Chen et al. likewise reported a higher proportion of blastocysts and improved implantation potential, but without differences in the total number of oocytes retrieved or fertilisation rate (20).

These discrepancies likely reflect methodological differences, including variation in study populations (general versus clinical), dietary assessment instruments (such as MEDAS-14 and MEDI-Lite), age distribution, and metabolic risk profiles (18). Despite this heterogeneity, studies reporting positive associations suggest that adherence to the Mediterranean Diet may influence ovarian health through mechanisms involving inflammatory modulation, metabolic regulation, and antioxidant protection (5, 7, 22).

### Endometriosis: evidence consistent with an anti-inflammatory role

The Mediterranean Diet appears to exert a protective effect against gynaecological conditions with an inflammatory basis, including endometriosis (28). Noormohammadi et al. (16) reported that higher adherence to healthy dietary patterns, assessed using MEDI-Lite and the Healthy Diet Indicator, was associated with a substantially lower likelihood of endometriosis (OR = 0.06; 95% CI 0.02–0.17). This association is linked to higher consumption of foods characteristic of this dietary pattern, including fruits, vegetables, legumes, and olive oil, as opposed to pro-inflammatory dietary components.

This body of evidence is reinforced by the analysis performed by Molina et al. (7) on the modulation of the endometrial metabolomic profile, supporting the hypothesis that the anti-inflammatory and antioxidant components of the Mediterranean Diet may be involved in the regulation of relevant pathophysiological pathways. In this context, these mechanisms may influence both the development of the condition and the severity of clinical symptomatology, as reported by Noormohammadi et al. (1, 16).

### Clinical and public health implications

The findings of this review reinforce the role of the Mediterranean Diet as a non-pharmacological dietary intervention with clinically relevant implications for female reproductive health. In clinical settings, adherence to this dietary pattern is associated with improvements in hormonal and metabolic profiles relevant to the management of polycystic ovary syndrome, which may occur independently of caloric restriction (2, 9). In assisted reproduction, preconceptional adherence has been linked to improved embryo quality and increased blastocyst formation, supporting its integration into preparation protocols for *in vitro* fertilisation (20).

Adherence to the Mediterranean Diet is also associated with a lower likelihood of gestational diabetes, potentially mediated through improved insulin sensitivity (22). Lifestyle interventions initiated prior to pregnancy have also been shown to reduce this risk by up to 25% (29).

Emerging evidence further supports the role of nutrition as a prognostic modulator of ovarian response, as pro-inflammatory dietary patterns may be associated with poor response even in the presence of normal ovarian reserve (8, 23). In this context, tools such as the Dietary Inflammatory Index enable identification of pro-inflammatory dietary profiles associated with increased infertility risk and a suboptimal ovarian microenvironment. Nutritional personalisation according to phenotype, including glycaemic control in polycystic ovary syndrome and anti-inflammatory strategies in endometriosis, may contribute to optimisation of both ovarian function and endometrial receptivity (14, 15).

From a public health perspective, these findings highlight the potential value of structured nutritional education programmes (21). Nevertheless, considering the observational nature and very low overall certainty of the current evidence, recommending the systematic implementation of dietary screening tools (such as MEDAS-14) in routine clinical practice appears premature. Future randomized controlled trials are required to establish their definitive clinical utility. Moving from passive dietary advice to structured, sustained interventions seem to be essential for ensuring adherence and maximising long-term metabolic and reproductive outcomes.

### Limitations

Several methodological limitations should be considered when interpreting our findings. First, most of the included studies used observational designs (cross-sectional and case–control), which limits the ability to establish definitive causal relationships. Consequently, according to the GRADE criteria, the overall certainty of the evidence for the association between the Dietary Inflammatory Index (DII) and female infertility was graded as very low. This classification is primarily driven by the observational nature of the included studies, which carries an inherent risk of residual confounding from unmeasured or suboptimally controlled variables. Although most primary studies adjusted for key confounding factors such as age and body mass index (BMI), factors such as total daily energy intake, physical activity levels, socioeconomic status, and pre-existing subclinical metabolic conditions could independently influence both dietary choices and reproductive health outcomes, making it difficult to isolate the specific effects of the Mediterranean diet.

Second, the quantitative synthesis is constrained by substantial imprecision resulting from the small number of pooled studies (*n* = 2) and a limited cumulative sample size.

The search strategy was restricted to studies published from 2021 onward to capture contemporary, post-pandemic clinical practice and to focus specifically on the recent integration of the Dietary Inflammatory Index (DII) in reproductive research. However, we explicitly acknowledge that this temporal restriction is a significant limitation, as it excludes a substantial body of earlier high-quality evidence that has fundamentally shaped current knowledge regarding Mediterranean diet adherence and female reproductive outcomes. This restriction directly explains the small number of eligible studies (*n* = 2) available for our quantitative meta-analysis. While our findings suggest a positive association between higher DII and infertility, the limited evidence base prevents a robust statistical assessment of publication bias, and these clinical implications must remain highly preliminary and cautious.

Additionally, while fully adjusted multivariable models comparing extreme DII categories were extracted from Wang et al. (23) and KabodMehri et al. (8) to ensure model comparability, subtle differences in residual covariate adjustments across primary studies remain a potential source of variance. Furthermore, the decision to exclude studies utilizing continuous E-DII metrics or self-reported subfertility definitions (e.g., Simon-Alesi et al.) was methodologically necessary to prevent clinical heterogeneity, though it restricted the final meta-analytic scope to two primary cohorts.

Furthermore, the concentration of approximately 80% of the statistical weight in a single study, namely Wang et al. (23), makes the pooled model highly dependent on one sample. Despite these constraints, the consistency in both the direction and magnitude of the effect across the available studies is compatible with a possible dose–response pattern, warranting further primary research to confirm whether a greater inflammatory potential of the diet is associated with a higher likelihood of infertility. As this is a rapidly evolving field where recent systematic reviews on dietary inflammatory potential have included substantially larger evidence bases, our quantitative findings should be viewed as preliminary, and clinical extrapolations must remain cautious.

Finally, the coexistence of different dietary indices and nutritional concepts within this review (including diet quality indices, specific Mediterranean scores, and the DII) represents a major methodological limitation. This conceptual diversity introduces an inherent difficulty in isolating and attributing the observed reproductive benefits solely to the structure of the Mediterranean Diet. While this issue has been addressed through a clear separation of the synthesis according to exposure and outcome variables, future clinical studies should prioritise the standardized and homogeneous use of specific tools (such as the MEDAS questionnaire) to discern whether the observed benefits depend specifically on the Mediterranean dietary pattern as a whole or, more generally, on any dietary pattern that successfully reduces the systemic dietary inflammatory load.

This meta-analysis indicates that a higher Dietary Inflammatory Index is associated with a 70% increase in the likelihood of female infertility (pooled OR = 1.70; 95% CI 1.23–2.34; *p* = 0.0013), with no evidence of between-study heterogeneity (*I*^2^ = 0%), reflecting a high degree of consistency across the included studies. However, these findings should be interpreted in light of important methodological limitations, including the small number of available studies (*n* = 2), which precludes robust assessment of publication bias, and the concentration of approximately 80% of the statistical weight in a single study, namely that by Wang et al. (23).

## Conclusion

This systematic review and meta-analysis highlight inverse observational associations between dietary inflammatory potential (DII) or Mediterranean Diet adherence and adverse female reproductive outcomes. Given that the evidence base consists almost exclusively of observational designs, these findings must be strictly interpreted as epidemiological associations rather than established causal mechanisms or therapeutic interventions.

The qualitative synthesis indicates that higher adherence to this dietary pattern is associated with a reduced likelihood of impaired ovarian function (with individual estimates ranging from OR 0.29 to 0.39 in the evaluated studies). This association is reflected in higher levels of anti-Müllerian hormone and greater antral follicle count, suggesting a beneficial influence on ovarian reserve and the follicular microenvironment.

Beyond ovarian reserve, the available literature demonstrates observational links between higher Mediterranean Diet adherence and lower risk or severity of gynaecological conditions with inflammatory and metabolic components, including polycystic ovary syndrome and endometriosis. In women with polycystic ovary syndrome, adherence to this dietary pattern is associated with improvements in insulin sensitivity and androgen balance. In endometriosis, higher adherence is associated with a markedly lower likelihood of the condition and reduced symptom severity.

In the context of assisted reproduction, qualitative findings cautiously suggest that the Mediterranean Diet might help preserve follicular potential and uterine receptivity. Its preliminary implementation during the preconception window has been observationally associated with higher blastocyst formation rates, improved embryo quality, and increased implantation rates, though these potential clinical benefits require robust experimental confirmation. While these observational trends align with proposed physiological mechanisms involving systemic inflammation and metabolic regulation, interventional clinical trials are strictly required to verify whether dietary modification exerts direct therapeutic efficacy and to assess the clinical utility of assessment tools such as DII or MEDAS-14.

From a public health perspective, the findings point to the need for more structured and personalised nutritional education programmes within primary care and gynaecological settings. While preliminary quantitative data suggests that pro-inflammatory dietary patterns (reflected in higher DII scores) could be associated with an increased risk of infertility, the extremely limited evidence base dictates that these findings be viewed with caution to avoid disproportionate clinical extrapolations. Concurrently, qualitative evidence shows that higher adherence to the Mediterranean Diet correlates with lower observed rates of gestational metabolic complications, such as gestational diabetes, though causal preventive claims cannot be inferred from observational data.

Overall, dietary patterns appear to play a meaningful role in reproductive health. These findings suggest that dietary patterns may play a meaningful role in reproductive health, though more robust, direct experimental evidence is required before specific nutritional screening protocols can be formally integrated as a central component of standard reproductive healthcare.

## Data Availability

The original contributions presented in the study are included in the article/Supplementary material, further inquiries can be directed to the corresponding author.
